# Open source tool for prediction of genome wide protein-protein interaction network based on ortholog information

**DOI:** 10.1186/1751-0473-5-8

**Published:** 2010-08-04

**Authors:** Chandra Sekhar Pedamallu, Janos Posfai

**Affiliations:** 1 New England Biolabs, Ipswich, MA, USA

## Abstract

**Background:**

Protein-protein interactions are crucially important for cellular processes. Knowledge of these interactions improves the understanding of cell cycle, metabolism, signaling, transport, and secretion. Information about interactions can hint at molecular causes of diseases, and can provide clues for new therapeutic approaches. Several (usually expensive and time consuming) experimental methods can probe protein - protein interactions. Data sets, derived from such experiments make the development of prediction methods feasible, and make the creation of protein-protein interaction network predicting tools possible.

**Methods:**

Here we report the development of a simple open source program module (*OpenPPI_predictor*) that can generate a putative protein-protein interaction network for target genomes. This tool uses the orthologous interactome network data from a related, experimentally studied organism.

**Results:**

Results from our predictions can be visualized using the *Cytoscape *visualization software, and can be piped to downstream processing algorithms. We have employed our program to predict protein-protein interaction network for the human parasite roundworm *Brugia malayi*, using interactome data from the free living nematode *Caenorhabditis elegans*.

**Availability:**

The *OpenPPI_predictor *source code is available from http://tools.neb.com/~posfai/.

## Introduction

The cell is the structural and functional unit of living organisms. Cells carry out numerous functions, from DNA replication, cell replication, protein synthesis, and energy production to molecule transport, to various inter- and intra-cellular signaling. Many of these fundamental processes require cascades of biochemical reactions that are catalyzed by interacting protein enzymes. Other interacting proteins provide structural support for the cells, form scaffolds for intracellular localization, and serve as chaperones or as transporters. The large-scale study of all cellular proteins is known as proteomics [1,2]. Since aspects of protein function can be inferred from the protein's complex interactions, from its position in interaction networks, one of the main goals of proteomics is to map the interactions of proteins. Uncovering protein-protein interaction information is a major undertaking in basic biological research, helps in the discovery of novel drug targets for the treatment of various diseases. Interaction networks (interactomes) for many model organisms have been established experimentally. Experimental probing of protein-protein interactions requires labor-intensive techniques, such as co-immunoprecipitation, or affinity chromatography [3]. High-throughput experimental techniques, such as yeast two-hybrid screens [4] and mass spectrometry [5] are also available for large-scale detection of protein-protein interactions, for the exploration of protein's amino acid sequences, their structures, and relationships [3]. Following these advances, numerous computational methods have been developed to predict protein-protein interaction networks. These use or combine phylogenetic profiling [6], homologous interacting partner analysis [7], structural pattern comparisons [8-10], bayesian network modeling [11], literature mining [12], codon usage analysis [13], and so on. Surveys on computational methods for prediction of protein-protein interactions are available in the literature [3,14]. Complementing efforts centralize protein-protein interaction data through the construction of databases, such as *STRING *[15], *MINT *[16], *BioGRID *[17], *DIP *[18], *POINT *[19] and *IntAct *[20].

Most of these reviewed prediction methods are implemented as web servers, which are convenient for the in-depth analysis of selected nodes and features, but offer little flexibility when the prediction of a complete cellular interactome is an intermediate goal, embedded in an involved discovery scheme. In this paper, we report the development of a simple open source tool (*OpenPPI_predictor*) for such intermediate role. The tool predicts the complete protein-protein interaction network for target genomes, using interactome data from related organisms (i.e. reference genomes). For further analysis, the generated putative interactome can be visualized using the *Cytoscape *software [21], and can be forwarded to follow-up program modules. We have developed this program to predict the protein-protein interaction network of the human parasite *B. malayi*. The predictions rely on the available interactome data of the close relative nematode *C. elegans*. The predicted number of interactions, and types of interacting partners, the distinguishing features from the human interactome guide our wet lab researchers in the selection of protein targets which seem essential for the parasite, so blocking them would disrupt its cell cycle, yet the intervention would not interfere with human protein complexes.

## Design and Implementation

This tool comprises of two modules: (a) Ortholog (diverged from the same immediate ancestor) protein identifier, and (b) Protein - Protein interaction predictor. The tool requires four kinds of inputs:

i. Sequences of proteins from the reference genome,

ii. Interactome for the reference genome (also called as orthologous interactome),

iii. Sequences of proteins from the genome of interest.

iv. Protein ortholog assignments between organism of interest and reference organism.

The ortholog protein identifier extracts information from an ortholog database, and makes connections across the reference genome and the genome of interest. The output from this module is a list of connections between proteins in the genome of interest and their corresponding orthologous relatives in the reference genome.

The protein - protein interaction predictor module uses the already known interactome of the reference genome. Interactions in the reference set are projected back to the corresponding orthologous proteins of the genome of interest.

More formally, the workflow of our method is as follows: assume we have two query proteins Q1 and Q2, with corresponding orthologous proteins R1 and R2 in the reference genome. If R1 and R2 interact in the reference organism, then the prediction is made, that Q1 and Q2 also interact. Knowledge about the relationship of R1 and R2 are transferred to a predicted relationship between Q1 and Q2.

**Figure 1:**
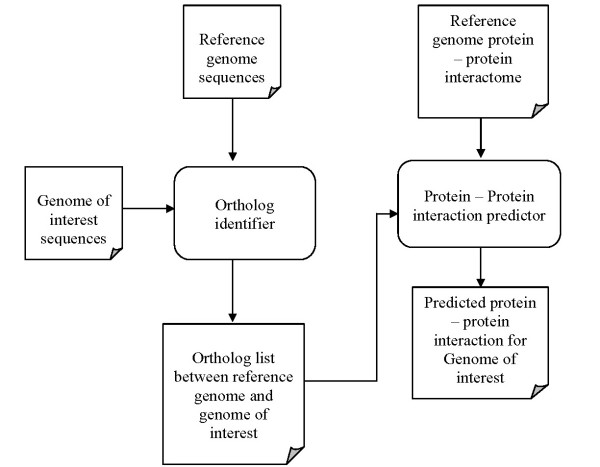
A block diagram describing the pipeline implemented *OpenPPI_predictor*

Figure 1 describes the overall implementation in *OpenPPI_predictor *tool. The algorithm used in this pipeline is divided into following steps:

Step 1: Create the ortholog connections between reference genome and genome of interest sequence, using ortholog identifier component from *OrthoMCL-DB*.

Step 2: Use protein-protein interaction predictor to predict interactome for genome of interest from interaction data in the reference genome, and the ortholog connections created in Step 1. The predicted interactome is in format that is compatible to *Cytoscape *software.

Step 3: Use *Cytoscape *software to visualize and analyze the predicted interactome generated in Step 2.

*OpenPPI_predictor *is implemented in AWK and C shell language and installed on Linux. The source code can be downloaded from http://tools.neb.com/~posfai/. The program has been tested by generating predictions for pairs of yeast and mammalian model organisms, using several resources listed in the Introduction.

## Results and Discussion

We have used *OpenPPI_predictor *to predict the *B. malayi *protein - protein interaction network. Genome and proteome data was fetched from NCBI (http://ncbi.nlm.nih.gov) ortholog assignments from *OrthoMCL-DB *[22], while reference *C. elegans *interactome data was downloaded from Worm Interactome Database [23].

The filarial nematode *B. malayi *is a human parasite. It causes elephantiasis, a wide spread and devastating disease, characterized by swelling of the lower limbs. Other filarial parasites, *Wuchereria bancrofti *and *Brugia timori *are also widespread, and cause serious diseases. Though these latter organisms differ from *B. malayi *morphologically, symptomatically, and in geographical extent [24], our target selection method can be followed in their cases as well. *C. elegans *is a free living nematode, and one of the most studied organisms, with available experimental genome, proteome, and interactome data. *C. elegans *interactome is used here to predict the *B. malayi *interactome, because of the high level of genomic conservation between these species [25]. The *C. elegans *interactome is composed of 178151 interactions, from the 20100 proteins encoded in the genome. The interactions have been established through large scale projects using different methods.

Orthology resources typically employ all-versus-all BLASTP analysis (Washington University, http://blast.wustl.edu), followed by some form of clustering (Jaccard clustering, bidirectional best hit clustering; [25]). Some tools, including *ProGMap *[26], *Berkeley PHOG *[27], *TdrTargets *[28], *BLASTO *[29], use additional, complementing sequence and structural information to identify orthologs across multiple organisms. Several ortholog databases have been compiled, using variants of the above procedures. For ortholog information between *C. elegans *and *B. malayi *genome we considered the Clusters of Orthologous Groups of proteins (*COG*s, [30]), and the Princeton Protein Orthology Database (*P-POD*, [31]), but settled on the more up-to-date and more accessible *OrthoMCL-DB *database ([22], http://www.orthomcl.org/common/ downloads/).

**Figure 2:**
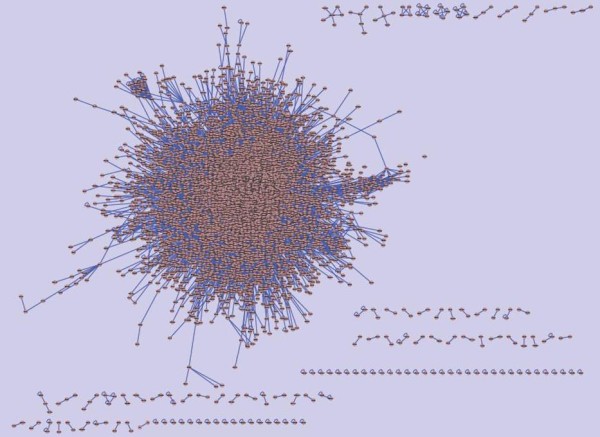
*C. elegans *interactome

**Figure 3:**
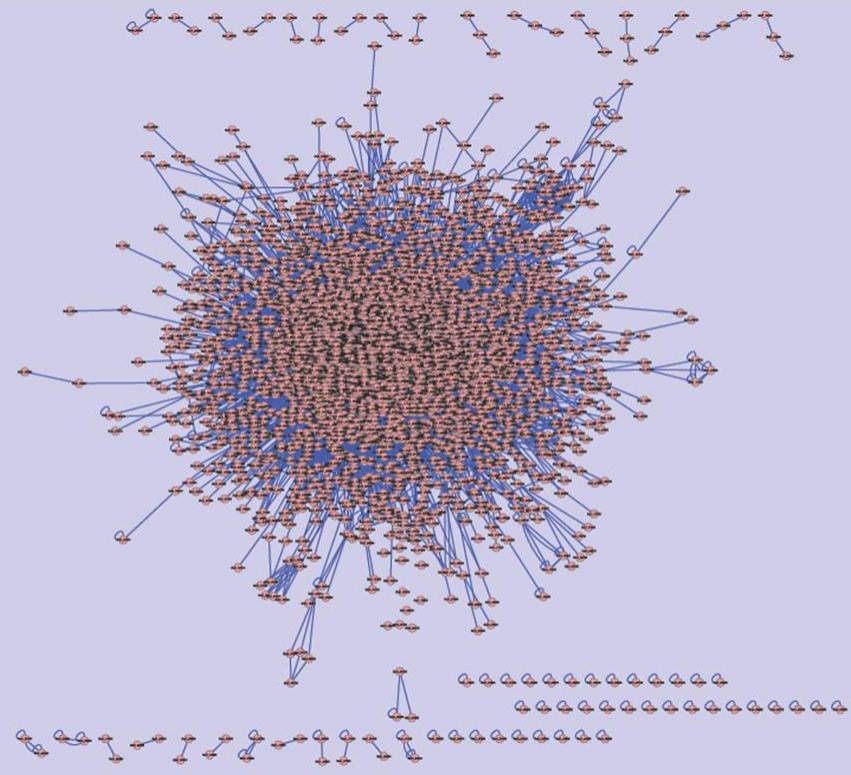
Predicted *B. malayi *interactome

Figure 2 and Figure 3 illustrate the *C. elegans *interactome and the predicted *B. malayi *interactome, using *Cytoscape *software. The predicted *B. malayi *interactome is composed of 164187 interactions from 11460 protein coding sequences. From our predictions, the *B. malayi *interactome seems sparser then the *C. elegans *interactome. This difference may be due to the fact, that *B. malayi *is a parasite, which exploits a host organism, hijacks some of its functions, metabolites, and processes. Incompleteness of the *B. malayi *genome sequence, and also the limited accuracy in the identification of ortholog relationships across *C. elegans *and *B. malayi *may contribute to sparseness.

**Figure 4:**
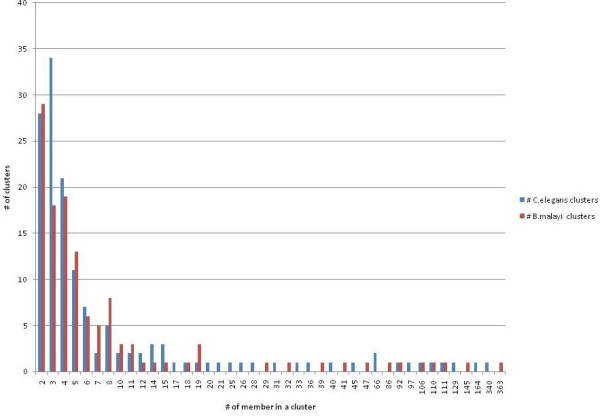
Distribution of clusters and number of cluster members

For a post-prediction analysis, we have used *Mcode *[32] to find clusters (highly connected regions) in the interaction network. Such clusters often correspond to protein complexes, and are parts of distinct metabolic pathways. *Mcode *identifies 118 and 143 clusters in *B. malayi *and *C. elegans *interactomes, respectively. The highly connected region contains 363 and 340 proteins in *B. malayi *and *C. elegans *interactome. This observation suggests that core cellular functions of the two related organisms have similar complexity. Figure 4 illustrates the distribution of clusters and number of cluster members. Further analysis of these highly connected regions may provide clues about genes missing from a conserved pathway, or proteins missing from a complex. The predicted interactome could be used to attribute protein function as well [33-35].

The utility of predictions depends on several factors. The establishment of orthology (relatedness through descent from the same common ancestor) carries less uncertainty, if a closely related reference organism can be found. Data from multiple related reference organisms would increase the signal to noise ratio, and improve the value of predictions. Experimentally verified test tube interactions may not be projected unconditionally to in vivo conditions: *i*. proteins interacting in a screen may not co-exist or co-localize in the living cell, they may be synthesized in different phases of the cell cycle, or they can be transported to different intracellular compartments, *ii*. post-translationally modified, mutated, alternatively spliced proteins may not interact with the same partners, *iii*. presence and binding of co-factors can change protein structure, hence interaction partnerships, *iv*. quorum signals can turn on and off interactions in bacteria, *v*. cell type and expression levels can modify interactions, *vi. *non-binary effects appear. Since the prediction tool uses such uncertain data, we should expect a degree of uncertainty in our predictions, and the results should be considered putative.

## Conclusions

Here we report the development of the *OpenPPI_predictor *tool. The tool predicts the protein interactome for a genome of interest, using the interactome data from a closely related organism, and protein orthology information between the two species. The tool is designed for genome wide interactome predictions, and provides a simple, flexible and easy to use platform for proteomic research.

Making predictions about possible protein-protein interactions is only an intermediate step in understanding protein function or in the search of drug targets. Upstream and downstream steps, biochemical and physiological considerations (many listed in earlier paragraphs) in finding applicable datasets, in filtering input data, in interpreting results and in drawing inferences make only the predictions relevant.

In the future, we plan to enhance both the utility and the coverage of our predictions using data from multiple related organisms, taking into account the phylogenetic distances between the interrogated pairs. We plan on ranking, or categorizing the predicted interactions according the consistency with which the predictions appear in the pair-wise predictions.

## Competing interests

The authors declare that they have no competing interests.

## Authors' contributions

CSP wrote source code for *openPPI_predictor*. CSP and JP wrote the manuscript. All authors have read and approved the final manuscript.
